# Efficacy and safety of cold snare polypectomy for outpatient treatment of sessile polyps smaller than 10mm

**DOI:** 10.1186/s12876-025-04245-8

**Published:** 2025-09-02

**Authors:** Chunmei Li, Xinyu Xie, Jian Qin, Yufei Ding, Xiaojuan Ma, Shanshan Liu, Miao Chen, Dandan Dong, Jing Sun, Xuedan Deng, Lulu Liu, Hongyan Cui

**Affiliations:** 1 Department of Oncology, The Fourth Division Hospital of Xinjiang Production and Construction Corps, Yining, China; 2 Department of Gastroenterology, The Fourth Division Hospital of Xinjiang Production and Construction Corps, Yining, China; 3 Department of Pathology, The Fourth Division Hospital of Xinjiang Production and Construction Corps, Yining, China; 4 Department of Internal Medicine, The Fourth Division Hospital of Xinjiang Production and Construction Corps, 62nd Regiment Hospital, Yining, China

**Keywords:** Colorectal polyp, Cold snare polypectomy, Optical assessment, Resection rate, Complete resection

## Abstract

**Background:**

Screening colonoscopy plays a critical role in reducing colorectal cancer incidence by identifying and removing polyps. Simple and safe treatment is the most common request of both doctors and patients. Cold snare polypectomy (CSP) is increasingly favored for polyps < 10 mm, yet concerns remain regarding residual tissue. The aim of this study was to evaluate the efficacy and safety of CSP in the outpatient treatment of sessile polyps < 10 mm using endoscopic optical assessment.

**Methods:**

Patients undergoing outpatient screening colonoscopy who consented to combined polypectomy were recruited, excluding those on anticoagulants or antiplatelets. CSP was performed for detected sessile polyps < 10 mm, and patients did not undergo any additional screening laboratory tests. Postoperative wounds were assessed endoscopically, and resected specimens were stained with crystalline violet for optimum pathological preparation and evaluation. Complete resection was determined separately. Complications and 7-day postoperative outcomes were recorded.

**Results:**

A total of 194 sessile colorectal polyps < 10 mm were resected from 77 patients, with a complete resection rate of 91.24% (95% confidence interval: 87.2–95.2%). There was a statistically significant difference in the rate of complete resection by endoscopic optical assessment compared to pathologic assessment (86.60% [168/194] vs. 72.68% [141/194], p < 0.01). Optical assessment was not significantly different from the final total resection rate (86.60% [168/194] vs. 91.24% [177/194], p = 0.15). No adverse events occurred in all patients.

**Conclusions:**

CSP is a safe and effective technique for outpatient resection of sessile polyps < 10 mm. Optical assessment of postoperative defects provides a viable method for determining complete resection.

**Trial registration:**

Trial registered at the Chinese Clinical Trial Registry (registration number: ChiCTR2400082461, registration date: 29/3/2024).

## Introduction

Colorectal cancer (CRC) is a leading gastrointestinal malignancy, with colorectal polyps recognized as key precancerous lesions. Screening colonoscopy is recommended as it reduces CRC-related mortality [1] by enabling early detection and endoscopic removal of adenomatous polyps, thereby interrupting the adenoma-to-carcinoma progression. More than 90% of colonoscopically detected polyps are < 10 mm [2–4]. Cold snare polypectomy (CSP) is widely used due to its simplicity, cost-effectiveness, and low complication rate. The European Society of Gastrointestinal Endoscopy (ESGE) recommends CSP for sessile polyps < 10 mm [5].However, concerns persist regarding residual tumor due to specimen retrieval failure, inaccurate margin assessment, and the absence of cauterization compared with hot polypectomy or endoscopic mucosal resection (EMR).Incomplete resection has been linked to interval CRC in 19–30.8% of cases [6]. Previous studies have evaluated CSP resection completeness through biopsies at the defect or margin [7], secondary EMR of the surrounding mucosa [8, 9], or retrospective biopsy of the postoperative scar after 3 weeks [10]. However, the limitations of pathology sampling of postoperative wounds may not be sufficient to assess the effectiveness of CSP. As the ‘resect and discard’ strategy is being discussed and promoted [11], real-time endoscopic diagnosis is of particular importance.

To address this limitation, we spread and stained postoperative CSP specimens with crystalline violet to facilitate precise margin identification, prepared pathological specimens, and conducted histopathological evaluation. Complete resection was assessed using endoscopic white-light and image-enhanced, and the results were compared with the pathologic diagnosis and final analysis of complete resection rates. Surgery-related complications and adverse events were also documented. To our knowledge, no prior studies have evaluated CSP efficacy using this approach. This single-center prospective study aimed to assess the efficacy and safety of CSP for sessile polyps < 10 mm in an outpatient setting, focusing on optical assessment of resection margins.

## Patients and methods

### Study design and patients

**Fig. 1:**
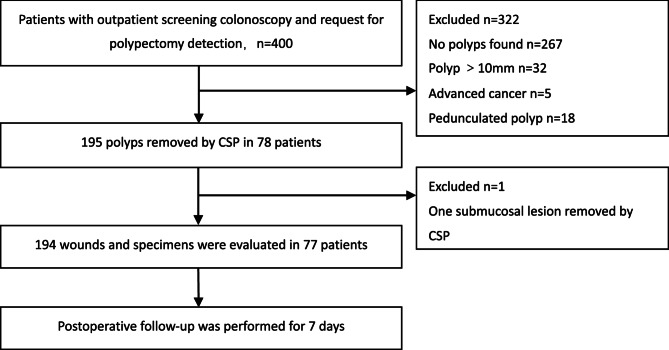
Flow chart showing patients enrollment and study design

This prospective, single-arm observational study included patients who underwent screening colonoscopy in an outpatient clinic and requested endoscopic treatment for colorectal polyps. Patients meeting the inclusion criteria underwent CSP on an outpatient basis. Macroscopic classification of polyps was based on the Paris classification [12]. Postoperatively, white-light and image-enhanced endoscopic evaluations were performed to assess the resection site. The specimens were flattened, stained with crystalline violet to identify the nearest incision edge, and processed for histopathological evaluation. Complete resection was assessed separately. Patients were followed up via telephone for 7 days to monitor complications (Fig. 1).

Between April 2024 and January 2025, patients from the Department of Gastroenterology at the Fourth Division Hospital of Xinjiang Production and Construction Corps were enrolled. Multiple polyps in a single patient were removed using the same snare. Inclusion criteria: (i) No use of anticoagulant or antiplatelet drugs, or discontinuation for > 5 days; (ii) Age ≥ 18 years; (iii) Consent for immediate polyp removal upon detection; (iv) Sessile colorectal polyps < 10 mm (Paris type Is, IIa, IIb). Exclusion criteria: (i) Pregnancy; (ii) Ulcerative colitis; (iii) Polyps ≥ 10 mm; (iv)Pedunculated polyps (Paris type Ip, Isp); (v) Polyps endoscopically assessed as cancerous. The study protocol, including a 7-day postoperative follow-up, was clearly explained to all patients, and written informed consent was obtained before enrollment.

The study was approved by the Medical Ethics Committee of the Fourth Division Hospital (registration number: BTSSHEC-C-2024-001-10) and conducted in accordance with the Declaration of Helsinki. It was registered with the Chinese Clinical Trial Registry (registration number: ChiCTR2400082461, registration date: 29/3/2024).

### Procedures

**Fig. 2:**
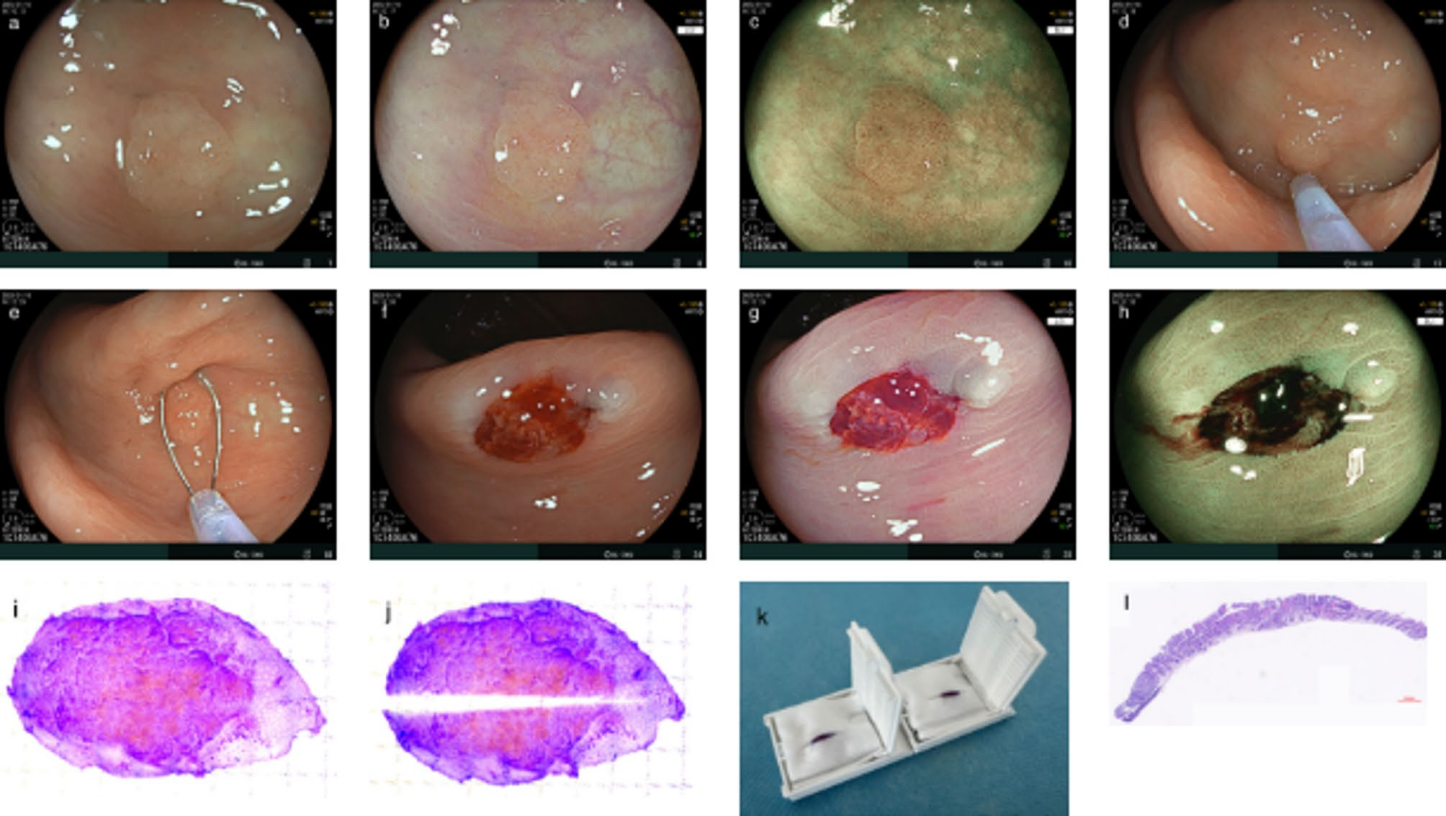
A case of CSP resection. **a** Polyp observed under white-light. **b** Polyp observed with linked color imaging (LCI). **c** Polyp observed with blue laser imaging (BLI). **d** Polyp size was assessed based on the diameter of the outer sheath of the snare. **e** Polyp containing normal mucosal tissue was resected. **f** White-light observation of the cold snare defect. **g** Linked color imaging (LCI) observation of the cold snare defect. **h** Blue laser imaging (BLI) observation of the cold snare defect. **i** Specimen stained with crystal violet after being laid flat. **j** Specimen cut but not completely dissected. **k** Postoperative specimens embedding. **l** Pathological image

Bowel preparation began the day before the procedure with a low-residue diet; red or seeded fruits were prohibited. Patients consumed 2000 mL of polyethylene glycol solution the night before and on the morning of the colonoscopy. Upon reaching the cecum, polyp location, size, and morphology were recorded during the retreat. Polyps were assessed under white-light and image-enhanced endoscopy, with histological classification determined by the NICE type [13] (Fig. 2a-c).

All CSP procedures were performed without the use of distal attachment caps. Polyps classified as NICE type 1 or 2 and meeting outpatient resection criteria were removed with CSP. The polyp was positioned at the 6 o’clock field of view, and standardized CSP was performed, ensuring removal of 1–2 mm of surrounding normal mucosa (Fig. 2e). Observe the wound and record the presence of protrusion within the cold snare defect(CSDP), immediate bleeding, and hematoma formation. Resected specimenswere collected via suction through the working channel of the colonoscope onto a placed gauze suction port. The CSP defect was further evaluated using high-definition white-light and image-enhanced endoscopy. A water jet was used to rinse the area affected by the bleeding to check for residual tumor tissue (Fig. 2f-h). Additional resections were performed if necessary. Endoscopic clips were applied if post-procedural bleeding exceeded 60 s.

After the operation, the patient went home on his own with instructions to seek medical attention if they experienced rectal bleeding or abdominal pain. Follow-up calls were made after 7 days.

Polyp size was estimated using the snare sheath diameter (Fig. 2d). All procedures were performed by a single experienced endoscopist with over 2, 000 colorectal polypectomies. CSP was performed using the Jiuhong snare device (230-cm length, 15-mm snare size, 0.24-mm snare wire diameter, 2.3-mm snare tip cup diameter; Jiuhong Medical Instrument Co., Ltd., China).

### Specimen processing

After completion of all polypectomy procedures in one patient, specimens were processed, flattened and fixed with formalin. CSP specimens were carefully unfolded, placed on a plastic plate, and stained with crystalline violet. The presence of lesion tissue at the margins was assessed (Fig. 2i). When the lesion was near the incision edge, a tangent line closest to the margin was identified, and samples were obtained at 2–3 mm intervals perpendicular to it. The specimen was then sectioned without disrupting tissue integrity, marked, imaged, and finally dissected (Fig. 2j). A sponge sheet with a central incision was placed inside the embedding cassette and each specimen was placed vertically in the incision (mucosal side perpendicular to the bottom of the cassette) to ensure correct orientation of the specimen for histological assessment (Fig. 2k). Tissue samples were analyzed with hematoxylin and eosin (H&E) staining by two blinded gastrointestinal pathologists in accordance with World Health Organization (WHO) standards [14].

### Definitions

After CSP, the cold snare defect was evaluated optically. The peripheral mucosa without lesion tissue was classified as negative, while areas with residual lesion tissue were positive. Indeterminate cases were those in which margins could not be adequately assessed. Similarly, stained specimens were categorized as negative if surrounded by normal mucosa, positive if lesion tissue extended to the margins, and indeterminate if proximity to the cut edge prevented a conclusive assessment. Tissue was assessed pathologically after H&E staining. Intact specimens and pathological sections containing more than two normal glandular ducts outside the lesion were classified as the negative group (Fig. 2l). Specimens fragmented, not sectioned perpendicular to the mucosal surface, or lacking normal glandular ducts outside the lesion were classified as the indeterminate group. A protrusion within the cold snare defect (CSDP) was defined as an elevated bundle of tissue observed immediately after CSP. Fragmented specimens referred to those appeared broken post-excision. The right colon included the cecum, ascending colon, and transverse colon, while the left colon comprised the descending and sigmoid colon.

Definition of adverse events: Intraoperative bleeding was defined as hemorrhage lasting > 60 s and requiring endoscopic intervention. Delayed bleeding referred to post-polypectomy bleeding occurring after 24 h.

### Sample size and statistical analysis

As this was an observational study, the sample size was randomized. A total of 120 polyp specimens were planned for statistical evaluation. The study showed that the adenoma detection rate (ADR) ranges from 14 to 15%, with the target ADR in the general population being ≥ 15% [15]. Based on prior institutional data, an average of two polyps per patient was expected. Thus, 400 patients undergoing outpatient colonoscopy were planned for inclusion.

Data were presented as rates or medians with interquartile ranges. Categorical variables were analyzed using Fisher’s exact test or the Chi-square test. Statistical significance was set at *P* < 0.05 (two-sided). Analyses were performed using SPSS Statisticssoftware, version 23.0 (SPSS Inc., IBM Corp., Armonk, NY, USA).

## Results

### Characteristics of patients and lesions

A total of 400 patients were initially included. Of these, 267 were polyp-free, 32 had polyps > 10 mm, 5 had advanced cancer, and 18 had only pedunculated polyps, leading to their exclusion. Additionally, one case involving a 3-mm polyp, later confirmed as a submucosal mesenchymal tumor after surgery, was excluded from the final analysis (Fig. 1). In the remaining 77 patients, 194 polyps (mean: 5.00 mm, interquartile range: 4.00–5.25 mm) were resected using CSP. All instances of intraoperative bleeding were limited to minor oozing and required no specific treatment. None of the resection sites required endoscopic clip placement. The characteristics of the 77 patients were summarized in Table 1.

**Table 1:** The characteristics of the 77 patients

Patient, *n*	77
Age, median (IQR), years	55 (48-61)
Sex	
female, *n* (%)	34 (44.16)
male, *n* (%)	43(55.84)

*IQR* Interquartile Range

Each polyp was removed in a single pass using CSP. Of the 194 lesions, 107 (55.15%) were located in the right colon, 67 (34.54%) in the left colon, and 20 (10.31%) in the rectum. Histopathological analysis revealed that 48 cases were hyperplastic polyps, 38 were sessile serrated lesions, and 2 were traditional serrated adenomas. Among the 106 adenomas, 104 were tubular adenomas, and 2 were tubulovillous adenomas. The complete resection rates assessed by endoscopic optics, specimen staining, and pathology were 86.60%, 88.14%, and 72.68%, respectively. The characteristics of the resected lesions were detailed in Table 2.

**Table 2:** Characteristics of colorectal polyps resected by CSP in the total sample

Polyps, *n*	194
size, median (IQR)	5.00 (4.00, 5.25)
Location, n (%)	
Cecum	16(8.25)
Ascending colon	42(21.65)
Hepatic flexure	16(8.25)
Transverse colon	31(15.98)
Splenicflexure	2(1.03)
Descending colon	17(8.76)
Sigmoid colon	50(25.77)
Rectum	20(10.31)
Morphology (Paris endoscopic classification, n (%)	
Is	11(5.67)
IIa	183(94.33)
Histopathological diagnosis, n (%)	
hyperplastic polyp	48(24.74)
Sessile serrated lesion	38(19.59)
Traditional serrated adenoma	2(1.03)
Tubular adenoma	104(53.61)
Tubulovillous adenoma	2(1.03)
Hematoma, n (%)	30(15.46)
CSDP, n (%)	33(17.01)
Specimen fragmentation, n (%)	10(5.15)
Complete resection rate, n (%)	
Optical assessment	168(86.60)
Staining assessment	171(88.14)
Pathological assessment	141 (72.68)
Complications	None

*IQR* Interquartile Range, *CSDP* Protrusion within the cold snare defect

**Fig. 3:**
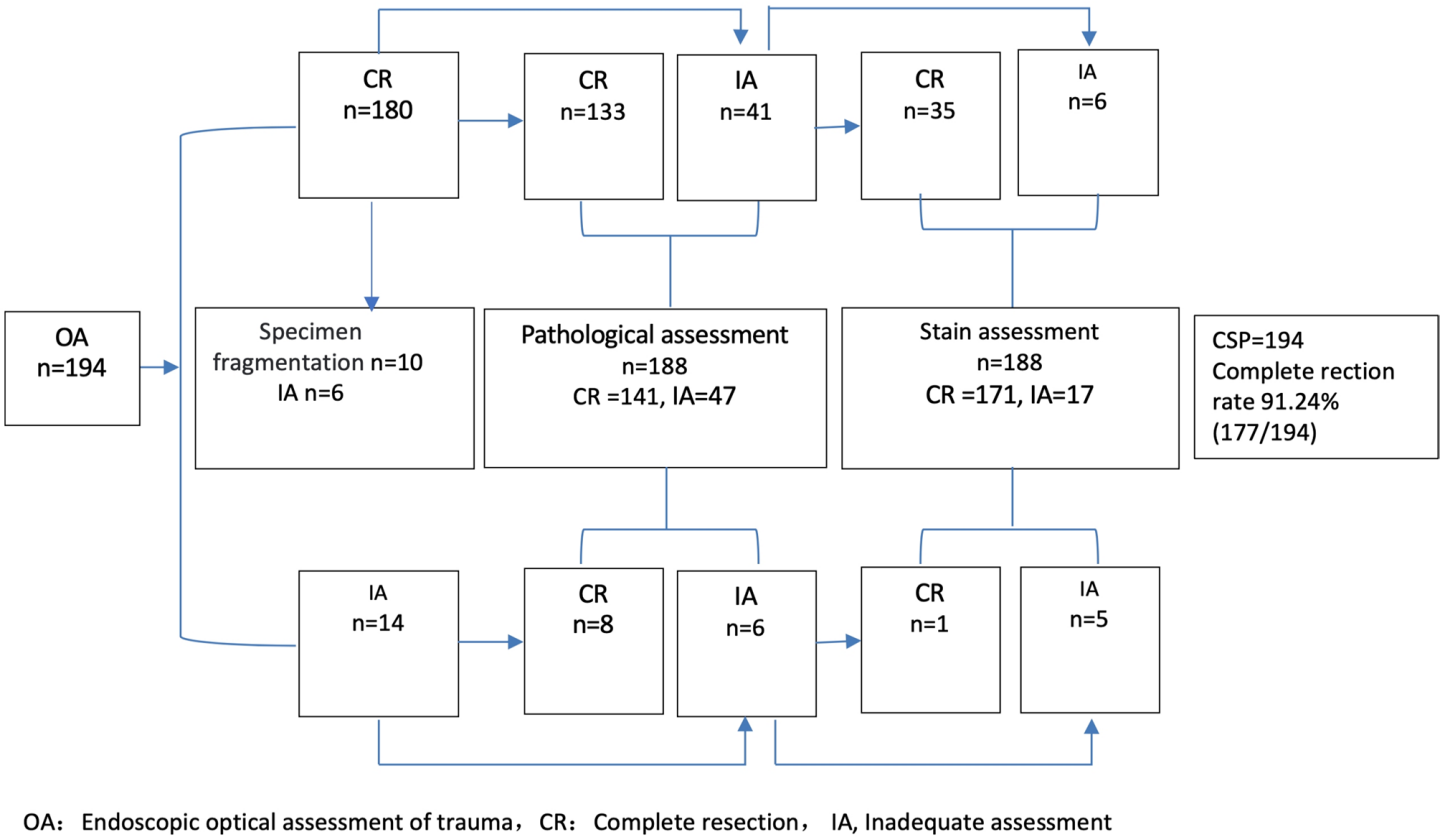
Postoperative evaluation flowchart

Ten specimens were fragmented postoperatively. Six cases of fragmentation occurred within the lesion were categorized as indeterminate, while the remaining four cases occurred outside the lesion were included in the subsequent statistical analysis. Endoscopic evaluation using white-light and image-enhanced endoscopy determined that 180 polyps had been completely resected. Pathological assessment confirmed complete resection in 133 cases. In 41 cases where pathology could not determine resection status, postoperative staining with crystal violet suggested complete resection in 35 cases, resulting in a total of 168 confirmed resections. Endoscopic evaluation was inconclusive in 14 cases; among these, pathology confirmed complete resection in 8 cases. Of the remaining 6 cases, postoperative staining indicated complete resection in 1 cases. Based on staining and pathologic evaluation, 177 polyps were confirmed to be completely removed. The final complete resection rate was 91.24% (95% CI: 87.2%−95.2%). The postoperative evaluation flowchart was presented in Fig. 3.

In summary, 194 colorectal sessile polyps < 10 mm were resected across 77 patients, achieving a complete resection rate of 91.24% (95% confidence interval, [CI]: 87.2–95.2%). There was a statistically significant difference in the rate of complete resection by endoscopic optical assessment compared to pathologic assessment (86.60% [168/194] vs. 72.68% [141/194], *p* < 0.01). No significant difference was observed between endoscopic optical assessment and final total resection rate (86.60% [168/194] vs. 91.24% [177/194], *p* = 0.15). Univariate and multivariate analyses showed no statistically significant differences in sex, age, polyp size and morphology, except for specimen fragmentation and location (Table 3).

**Table 3:** Analysis of reasons for inadequate assessment

	Complete resection (*n* = 177)		Inadequate assessment (*n* = 17)	Univariate analysis		Multivariate analysis	
				χ^2^	*P*	*P*	OR (95%CI)
Sex				0.034^a^	0.853		
Male		100	10				
Female		77	7				
Age (years)					0.477^b^		
≥ 65		28	1				
< 65		149	16				
Location				8.393^a^	0.015	0.009	3.567(1.366,9.314)
Left colon		66	1				
Right colon		95	12				
Rectum		16	4				
Size (mm)					0.999^b^		
>5		44	4				
≤ 5		133	13				
Morphology					0.603^b^		
IIa		166	17				
Is		11	0				
CSDP					0.742^b^		
Negative		146	15				
Positive		31	2				
Fragmentation					0.000^b^	0.000	18.175(4.552,72.566)
Negative		173	11				
Positive		4	6				
Hematoma					0.080^b^		
Negative		147	17				
Positive		30	0				

^a^Chi-square test

^b^Fisher^’^s exact test

## Discussion

This study is the first prospective evaluation of the safety and efficacy of CSP in outpatient screening colonoscopy patients with sessile colorectal polyps < 10 mm. All patients underwent polypectomy at the same time as outpatient screening colonoscopy, and no additional tests or labs were performed previously. The method involved flattening postoperative specimens, staining them with crystal violet, and obtaining high-quality pathological sections to determine whether polyps were completely excised. The results were compared with optical assessments. No significant difference in complete resection rates was observed between endoscopic optical and final total resection evaluations. The safety of CSP is reinforced by the absence of complications in both the immediate postoperative period and follow-up.

With the widespread use of screening colonoscopy, more and more polyps are being detected and removed endoscopically. Efficient, simple, and safe colonic polyp treatment procedure is a critical concern for clinicians. The ESEG strongly recommends CSP for treating sessile colorectal polyps < 10 mm but also emphasizes the need to include 1–2 mm of normal mucosa surrounding the polyp in the resection [5]. Incomplete polyp resection remains a significant concern for the occurrence of interval colorectal cancer [6]. A previous randomized controlled trial that included additional biopsies from the base and margins of the polypectomy site estimated the complete resection rate of CSP at 93.2% [7]. Another studies using EMR to excise surrounding mucosa at CSP sites reported a complete resection rate exceeding 95% [16, 17]. However, current procedural techniques still limit the adequacy of evaluation of CSP defects and surrounding mucosal pathology. Previous research has shown that the rate of unclear pathological margins in CSP postoperative specimens was 35.6% [17]. Although Ikeda et al. [18] demonstrated that pasting CSP specimens significantly improves margin assessment, the incidence of unclear margins is still as high as 15.1%.

As the number of polyp specimens removed by CSP increases, it becomes difficult to make an accurate pathological diagnosis due to specimen curling, fragmentation, and unclear margins. In our study, the pathological diagnosis complete resection rate was 72.68% (141/194). By reviewing and evaluating crystal violet staining images that could not be determined pathologically, the complete resection rate increased to 91.24%, which also highlights the challenges faced by pathologists. Endoscopic optics assessed 194 surgical wounds, of which 180 were assessed for complete resection and 14 were not assessable. Combined with crystal violet staining and pathological findings, 168 cases were confirmed as completely resected, yielding a complete resection rate of 86.60% (95% CI: 81.8–91.4%). No significant difference was found between endoscopic optical assessment and final complete resection rate (86.60% [168/194] vs. 91.24% [177/194], *p* = 0.15). This also confirms the validity of real-time optical assessment of the peri-wound mucosa after colorectal polypectomy. Both our final complete resection rate and optical assessment complete resection rate were slightly lower than previously reported. This is due to our strict definition of complete resection, which may improve the reliability of future studies and provide a new perspective for the comprehensive evaluation of CSP.

Maruoka et al. [10] resected 111 adenomas using CSP and marked the resected sites with clips. Pathological analysis showed that 32.4% of the adenomas were completely resected, but biopsies of the resected sites 3 weeks later revealed residual adenomatous tissue in only 0.98% of cases. This also suggests that pathologic evaluation of complete resection may not be effective in evaluating the efficacy of resection. Since the malignant rate of colorectal polyps smaller than 10 mm is very low, and the malignant rate of polyps smaller than 5 mm is even lower [19], the ‘resect and discard’ method may be appropriate. In this case, using optical examination to assess whether complete resection has been achieved is more convenient and feasible.

Immediate postoperative bleeding (IPB) and hematomas can blur endoscopic views, potentially hindering meticulous examination of the polypectomy site and increasing the risk of accidental incomplete resection. IPB has also been identified as a risk factor for polyp recurrence [20]. However, modern water injection devices facilitate bleeding clearance, improving the accuracy of optical assessment. Univariate analysis showed no significant correlation between hematomas and incomplete resection.

CSDP is sometimes observed after CSP and is generally considered a potential indicator of incomplete resection. Histopathological studies have identified mucosal muscular layer tissue in CSDPs [21]. When CSDPs are present, resected specimens often contain only small amounts of the mucosal muscular layer [22], contributing to specimen fragmentation [23]. In our study, 33 CSDPs were observed, of which 31 lesions were ultimately classified as completely resected. Ten postoperative specimens exhibited fragmentation. In four cases, the breakage occurred in the mucosal area outside the lesion and was assessed for complete excision, while the remaining six cases were categorized directly into the non-assessable group. The presence of specimen fragmentation and CSDP was not significantly associated with incomplete resection.

We used a non-dedicated snare for CSP, primarily because studies suggest that snare wire diameter does not significantly impact resection efficacy for small colorectal polyps [24]. Incomplete resection was also related to the endoscopist’s experience and level of operation [25, 26], so for this study we used the results of one operator. Location and specimen fragmentation showed statistically significant differences when comparing complete resection to inadequate evaluation. It may be related to the small amount of specimens, and to the fact that we categorized some of the fragmented specimens directly to the group that could not be evaluated. This needs to be assessed in the future with large samples.

This study has several limitations. First, it was conducted at a single institution, with all procedures performed by a single endoscopist, introducing potential bias. Second, the sample size was relatively small. Third, visual estimation of polyp size may have varied from actual size. Fourth, due to the small size of the specimens and to simplify the evaluation process, we did not use fine-needle fixation, which could have minimized specimen shrinkage and invagination of horizontal cutting edges. This methodological choice may have contributed to underestimation of the complete resection rate compared with previous studies.

## Conclusion

CSP is a safe and effective method for resecting sessile colorectal polyps < 10 mm. Even in outpatients, no additional laboratory tests or treatments were performed before, during, or after the procedure. Optical assessment of postoperative defects provides endoscopists with a simple and reliable method of assessing complete resection.

## Data Availability

The datasets used and/or analyzed during the current study are available from the corresponding author on reasonable request.

## Abbreviations

CSP: Cold Snare Polypectomy
CSDP: protrusion within the cold snare defect
IQR: Interquartile Range
IPB: Immediate Postoperative Bleeding
CR: Complete Resection
CI: Confidence Interval
IA: Inadequate Assessment

## References

[1] Nishihara R, Wu K, Lochhead P, et al. Long-term colorectal-cancer incidence and mortality after lower endoscopy. N Engl J Med. 2013;369(12):1095–105. 24047059 10.1056/NEJMoa1301969 PMC3840160

[2] Regula J, Rupinski M, Kraszewska E, et al. Colonoscopy in colorectal-cancer screening for detection of advanced neoplasia. N Engl J Med. 2006;355(18):1863–72. 17079760 10.1056/NEJMoa054967

[3] Hassan C, Pickhardt PJ, Kim DH, et al. Systematic review: distribution of advanced neoplasia according to polyp size at screening colonoscopy. Aliment Pharmacol Ther. 2010;31(2):210–7. 19814745 10.1111/j.1365-2036.2009.04160.x

[4] Gupta N, Bansal A, Rao D, et al. Prevalence of advanced histological features in diminutive and small colon polyps. Gastrointest Endosc. 2012;75(5):1022–30. 22405698 10.1016/j.gie.2012.01.020

[5] Ferlitsch M, Hassan C, Bisschops R, et al. Colorectal polypectomy and endoscopic mucosal resection: European society of Gastrointestinal endoscopy (ESGE) Guideline - Update 2024. Endoscopy. 2024;56(7):516–45. 38670139 10.1055/a-2304-3219

[6] Robertson DJ, Lieberman DA, Winawer SJ, et al. Colorectal cancers soon after colonoscopy: a pooled multicohort analysis. Gut. 2014;63(6):949–56. 23793224 10.1136/gutjnl-2012-303796 PMC4383397

[7] Lee CK, Shim JJ, Jang JY. Cold snare polypectomy vs. Cold forceps polypectomy using double-biopsy technique for removal of diminutive colorectal polyps: a prospective randomized study. Am J Gastroenterol. 2013;108(10):1593–600. 24042189 10.1038/ajg.2013.302

[8] Kim JS, Lee BI, Choi H, et al. Cold snare polypectomy versus cold forceps polypectomy for diminutive and small colorectal polyps: a randomized controlled trial. Gastrointest Endosc. 2015;81(3):741–7. 25708763 10.1016/j.gie.2014.11.048

[9] Matsuura N, Takeuchi Y, Yamashina T, et al. Incomplete resection rate of cold snare polypectomy: a prospective single-arm observational study. Endoscopy. 2017;49(3):251–7. 28192823 10.1055/s-0043-100215

[10] Maruoka D, Arai M, Akizue N, et al. Residual adenoma after cold snare polypectomy for small colorectal adenomas: a prospective clinical study. Endoscopy. 2018;50(7):693–700. 29415287 10.1055/s-0043-124869

[11] Rondonotti E, Hassan C, Andrealli A, et al. Clinical validation of BASIC classification for the resect and discard strategy for diminutive colorectal polyps. Clin Gastroenterol Hepatol. 2020;18(10):2357–e23654. 31923641 10.1016/j.cgh.2019.12.028

[12] The Paris endoscopic classification of superficial. Neoplastic lesions: esophagus, stomach, and colon: November 30 to December 1, 2002. Gastrointest Endosc. 2003;58(6 Suppl):S3–43. 14652541 10.1016/s0016-5107(03)02159-x

[13] Hewett DG, Kaltenbach T, Sano Y, et al. Validation of a simple classification system for endoscopic diagnosis of small colorectal polyps using narrow-band imaging. Gastroenterology. 2012;143(3):599–e6071. 22609383 10.1053/j.gastro.2012.05.006

[14] Nagtegaal ID, Odze RD, Klimstra D, et al. The 2019 WHO classification of tumours of the digestive system. Histopathology. 2020;76(2):182–8. 31433515 10.1111/his.13975 PMC7003895

[15] National Cancer Center, China, Expert Group of the Development of China Guideline for the Screening EDaEToCC. [China guideline for the screening, early detection and early treatment of colorectal cancer (2020, Beijing)]. Zhonghua Zhong Liu Za Zhi. 2021;43(1):16–38. 33472315 10.3760/cma.j.cn112152-20210105-00010

[16] Takeuchi Y, Yamashina T, Matsuura N, et al. Feasibility of cold snare polypectomy in japan: A pilot study. World J Gastrointest Endosc. 2015;7(17):1250–6. 26634041 10.4253/wjge.v7.i17.1250 PMC4658605

[17] Ichihara S, Uraoka T, Oka S. Challenges associated with the pathological diagnosis of colorectal tumors less than 10 mm in size. Dig Endosc. 2018;30(Suppl 1):41–4. 29658649 10.1111/den.13038

[18] Ikeda T, Yoshizaki T, Eguchi T, Kinugasa H, Okada A. Efficacy of specimen pasting after cold snare polypectomy for pathological evaluation of horizontal margins. Endosc Int Open. 2022;10(5):E572–9. 35571463 10.1055/a-1784-6723 PMC9106410

[19] Pooler BD, Kim DH, Matkowskyj KA, et al. Growth rates and histopathological outcomes of small (6–9 mm) colorectal polyps based on CT colonography surveillance and endoscopic removal. Gut. 2023;72(12):2321–8. 37507217 10.1136/gutjnl-2022-326970 PMC10822024

[20] Kuwai T, Yamada T, Toyokawa T, et al. Local recurrence of diminutive colorectal polyps after cold forceps polypectomy with Jumbo forceps followed by magnified narrow-band imaging: a multicenter prospective study. Endoscopy. 2019;51(3):253–60. 30674046 10.1055/a-0833-8548

[21] Tutticci N, Burgess NG, Pellise M, Mcleod D, Bourke MJ. Characterization and significance of protrusions in the mucosal defect after cold snare polypectomy. Gastrointest Endosc. 2015;82(3):523–8. 25910666 10.1016/j.gie.2015.01.051

[22] Shichijo S, Takeuchi Y, Kitamura M, et al. Does cold snare polypectomy completely resect the mucosal layer? A prospective single-center observational trial. J Gastroenterol Hepatol. 2020;35(2):241–8. 31389623 10.1111/jgh.14824

[23] Ishii T, Harada T, Tanuma T, et al. Histopathologic features and fragmentation of polyps with cold snare defect protrusions. Gastrointest Endosc. 2021;93(4):952–9. 32730821 10.1016/j.gie.2020.07.040

[24] Giri S, Jearth V, Darak H, Sundaram S. Outcomes of thin versus thick-wire snares for cold snare polypectomy: a systematic review and meta-analysis. Clin Endosc. 2022;55(6):742–50. 36347525 10.5946/ce.2022.141 PMC9726435

[25] Choi JM, Lee C, Park JH, et al. Complete resection of colorectal adenomas: what are the important factors in fellow training. Dig Dis Sci. 2015;60(6):1579–88. 25540087 10.1007/s10620-014-3500-0

[26] Arimoto J, Chiba H, Higurashi T, et al. Risk factors for incomplete polyp resection after cold snare polypectomy. Int J Colorectal Dis. 2019;34(9):1563–9. 31312890 10.1007/s00384-019-03347-6

